# First isolation and characterization of *Chryseobacterium shigense* from rainbow trout

**DOI:** 10.1186/1746-6148-8-77

**Published:** 2012-06-07

**Authors:** Leydis Zamora, Ana I Vela, Mª Angel Palacios, Lucas Domínguez, José Francisco Fernández-Garayzábal

**Affiliations:** 1 Centro de Vigilancia Sanitaria Veterinaria (VISAVET). Universidad Complutense, 28040 Madrid, Spain; 2 Departamento de Sanidad Animal. Facultad de Veterinaria, Universidad Complutense, 28040 Madrid, Spain; 3 Grupo Piszolla, S.L, 37800, Alba de Tormes, Salamanca, Spain

## Abstract

**Background:**

There have been an increasing number of infections in fish associated with different species of *Chryseobacterium*, being considered potentially emerging pathogens. Nevertheless the knowledge of the diversity of species associated with fish disease is partial due to the problems for a correct identification at the species level based exclusively on phenotypic laboratory methods.

**Results:**

*Chryseobacterium shigense* was isolated from the liver, kidney and gills of diseased rainbow trout in different disease episodes that occurred in a fish farm between May 2008 and June 2009. Identity of the isolates was confirmed by 16 S rRNA gene sequencing and phenotypic characterization. Isolates represented a single strain as determined by random amplified polymorphic DNA analysis.

**Conclusions:**

This is the first description of the recovery of *C. shigense* from clinical specimens in trout, a very different habitat to fresh lactic acid beverage where it was initially isolated.

## Background

Members the genus *Chryseobacterium* are widely distributed microorganisms that can be recovered from a wide variety of environments, such as fresh water, sewage and wastewater, soil or food sources, such as milk, poultry and meat and dairy products [1]. Some species of *Chryseobacterium* have been involved in human infections, acting as sporadic but severe opportunistic nosocomial pathogens [2,3]. In veterinary medicine, chryseobacteria are not relevant pathogens for domestic animals, but they are widely distributed in aquatic environments and fish farms [1,4]. Until recently members of the genus *Chryseobacterium* were not commonly associated with fish infections. However, there has been an increase in the frequency of clinical cases in which *Chryseobacterium* sp. strains have been isolated from different fish species. Thus, *Chryseobacterium balustinumChryseobacterium scophtalmum* and *Chryseobacterium joostei* have been isolated from diseased fish [4-6]. More recently, *Chryseobacterium piscicola* has been reported to produce mortalities in farmed Atlantic salmon (*Salmo salar*) and rainbow trout (*Oncorhynchus mykiss*) in Chile and Finland [7-9], *Chryseobacterium arothri* was isolated from the kidneys of the pufferfish *Arothron hispidus* in Hawaii [10] and *Chryseobacterium chaponense* from diseased farmed Atlantic salmon in Chile [11]. In fact, some *Chryseobacterium* species are considered potentially emerging pathogens in fish [4]. However, many chryseobacteria isolated from diseased fish are usually identified only at the genus level due to the difficulty of their correct identification by phenotypically based laboratory methods alone [4,5], which limits the knowledge of the diversity of species associated with fish disease.

## Methods

### Bacterial strains and culture conditions

The bacterial isolates were recovered from liver (635–08, 628-2-08; 692–08), kidney (664–09) and gills (706B-08, 972B-08, 1107B-09) of rainbow trout (*Oncorhynchus mykiss*) fry during five outbreaks (May, June and September of 2008 and June 2009) occurred in a fish farm located in the central region of Spain. The fish farm had a flow-through system with intake of water from an adjacent river. The water temperature is quite constant during the whole year (15 °C ± 1). Stocking densities vary along the growth period from 3 to 20 kg/m^3^, with a water exchange rate of 15–20 min. Feed consist in a commercial brand of extruded micropelets. Average feeding rate is about 3 % of biomass, delivered in four times by hand. Based on clinical symptoms and epidemiological background (*Flavobacterium psychrophilum* had previously been isolated from the farm) rainbow trout fry syndrome (RTFS) was suspected. Trout were submitted alive to the Animal Health Surveillance Centre (VISAVET) of the Universidad Complutense of Madrid for a confirmatory microbiological diagnosis. Trout were euthanized and necropsied under aseptic conditions. Samples of liver, kidney and gills were incubated on Anacker and Ordal´s agar for 7 days at 14 °C. Nutrient agar was used for routinely growth of clinical isolates after their initial isolation. Stock cultures were preserved at −80 °C in a cryopreservative media composed of tryptone (2.5 %), unskimed milk (5 %) and glicerine (20 %).

### F. psychrophilum PCR assay

The PCR assay specific for *F. psychrophilum* was performed as described by Wiklund et al. [12].

### 16 S rRNA gene sequencing

The 16 S rRNA gene of the seven isolates was amplified and sequenced as described previously [13] and subjected to a comparative analysis. A nearly complete 16 S rRNA gene fragment (>1,400 bp) was obtained bidirectionally using the universal primers pA (5’-AGAGTTTGATCCTGGCTCAG; positions 8–27, *Escherichia coli* numbering) and pH* (5’-AAGGAGGTGATCCAGCCGCA; positions 1,541-1,522, *E. coli* numbering). The determined sequences were compared with the sequences of other Gram-negative species available in the GenBank database, by using the FASTA program (http://www.ebi.ac.uk/fasta33). Phylogenetic relationships were inferred using the neighbor-joining algorithm as described previously [14].

### Random amplified polymorphic DNA fingerprinting

For all strains genomic DNA was prepared using method described by Marmur [15]. The primers used for RAPD-PCR were P1 (5’-CTGCTGGGAC-3’) and P2 (5’-CGCCCTGCCC-3’) (Roche Diagnostics S.L.) described previously (3). PCR amplifications were performed using a commercial PCR master mix (kit QIAGEN Multiplex PCR) adding the DNA template (5 μl), 0.5 μM of each primer and water up to a final volume of 25 μl. PCR amplifications were carried out in a Mastercycler gradient thermocycler (Eppendorf) with the following parameters: an initial denaturalization of 15 min at 95 °C and 30 cycles of 1 min at 94 °C, 1 min at 36 °C, and 2 min at 72 °C. PCR-amplified products (20 μl) were separated at 60 V for 2 h in 1.5 % agarose gel electrophoresis supplemented with 1X Syber safe® (Invitrogen, Eugene, OR). DNA banding patterns were analyzed using bioNumerics software (Applied Maths) to calculate Dice coefficients of correlation and to generate a dendrogram using the unweighted pair group method of arithmetic averages (UPGMA) clustering. To assess the repeatability of RAPD-PCR, isolates were submitted to three different amplifications assays for each primer, realized in different days and in similar conditions as described above.

### Phenotypic analysis

Isolates were characterized using conventional phenotypic tests proposed by Bernardet et al. [16] i.e. production of catalase and oxidase, motility, hydrolysis of agar, casein, L-tyrosine, aesculin, DNA, urea, gelatin and starch; production of flexirubin-type pigments; growth on MacConkey (bioMérieux) and nutrient (Difco) agars and growth at 15, 25, 30, 37 and 42 °C, with 3.0, 4.5 and 6.5 % added NaCl, and under anaerobic and micro-aerobic conditions, were determined as described previously [17]. The isolates were further biochemically characterized using the API 20NE and API ZYM systems (bioMérieux) according to the manufacturer’s instructions, except that incubation temperature was 25 °C. The type strain of *C. shigense* CCUG 61059^T^ = DSM 17126^T^ was used as a reference in all tests.

## Results

**Figure 1:**
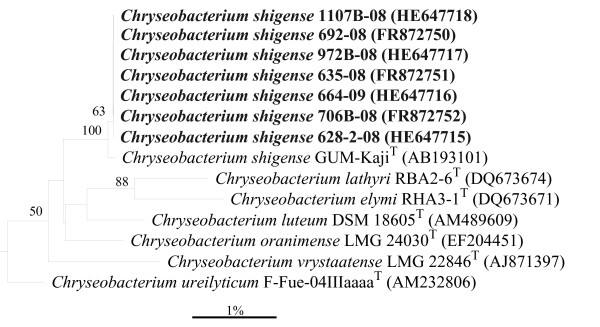
**Phylogenetic relationships of the clinically trout isolates and close related species in the genus*Chryseobacterium*inferred using the neighbor-joining method with 16 S rRNA gene sequences.** Bootstrap values (expressed as a percentage of 1000 replications) >50 % are given at the branching points. *Leeuwenhoekiella marinoflava* ATCC 19326^T^ (M58770) was used as outgroup. Bar, 1 % sequence divergence.

All isolates gave shiny, round, yellow-pigmented colonies on Anacker and Ordal agar, a characteristic that lead to the presumptive diagnosis of infection by *F. psychrophilum*, but none of the isolates gave the expected amplicon product of 1,089 bp, specific of *F. psychrophilum*. Moreover, cells of trout isolates were straight short Gram negative rods after Gram staining. Comparative analysis of the 16 S rRNA gene sequences revealed that the isolates shared 99.8-100 % sequence similarity between each other, thus demonstrating their high phylogenetic relatedness, 99.2-99.8 %, with the type strain of *C. shigense* (GUM-Kaji^T^; Figure 1) and only 81.8-81.9 % with *F. psychrophilum* NCIMB 1947^T^ (GenBank accession n D12670). The 16 S rRNA gene sequences of the isolates included in this study have been deposited in GenBank under the accession numbers indicated in Figure 1.

Phenotypically all trout isolates were catalase and oxidase positive, non-motile, grew on nutrient agar with yellow and shiny colonies but not on MacConkey agar, produced flexirubin-type pigment, were able to grow at 5–30 °C but not at 37 °C, and hydrolysed starch, casein and gelatin. With the API 20NE system they exhibited homogeneous biochemical characteristics displaying the numerical profiles 3452205, while *C. shigense* CCUG 61059^T^ gave the numerical profile 2456204. With the APY ZYM strips, the trout isolates, as well as the type strain of *C. shigense* CCUG 61059^T^, expressed activity for alkaline phosphatase, leucine arylamidase, trypsin, acid phosphatase and naphthol-AS-BI-phophohydrolase but not for esterase C4, lipase C14, cystine arylamidase, α-chymotrypsin, α-galactosidase, β-galactosidase, β-glucuronidase, α-glucosidase, *N*-acetyl-β-glucosaminidase, α-mannosidase and α-fucosidase. Clinical isolates of *C. shigense* expressed activity for valine arilamidase and not for ester lipase C8 and β-glucusidase, while *C. shigense* CCUG 61059^T^ gave opposite results for these tests.

**Figure 2:**
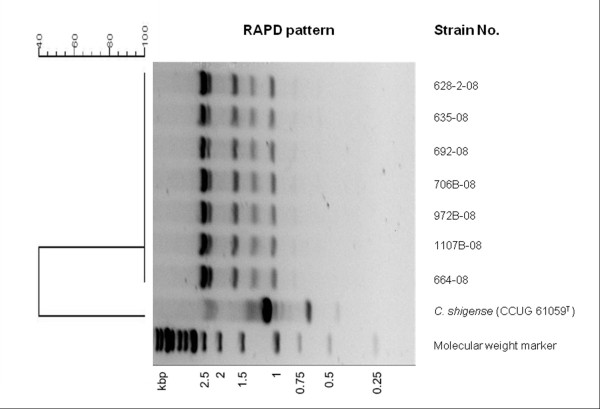
**RAPD profiles obtained with primer P2 for the*C. shigense*strains and the dendrogram generated based on the Dice coefficient using the BioNumerics software (Applied Maths).**

After genetic characterization by random amplified polymorphic DNA, both oligonucleotides generated reproducible patterns, but an appropriate number on bands were produced with oligonucleotide P2 (Figure 2). The seven *C. shigense* trout isolates showed undistinguishable RAPD fingerprints with amplifications bands ranging from 600 to 2500 bp, indicating genetic homogeneity among them. On the other hand, the strain CCUG 61059^T^ yielded a completely different fingerprint (Figure 2).

## Discussion

Diagnosis of bacterial fish diseases is not possible purely on the basis of the clinical signs and symptoms observed in diseased fish, because many of them can be caused by more than one etiological agent. Presumptive diagnosis is usually based on previous epidemiological data and a rough bacteriological analysis of cultured organisms from affected animals. In the present study, isolates were recovered from diseased trout suspected of *F. psychrophilum* infection because the fish farm had previous records of infections with this fish pathogen and the macroscopic characteristics of the colonies obtained on Anacker and Ordal agar were also compatible with that presumptive diagnosis. However, cell morphology of the trout isolates following Gram staining was different of the filamentous rods exhibited by *F. psychrophilum *[18], they were non-motile and none gave a positive reaction with a *F. psychrophilum* species-specific PCR assay [13]. This lack of amplification is consistent with the low similarity observed (81.8-81.9 %) between the 16 S rRNA gene sequences of trout isolates and *F. psychrophilum* NCIMB 1947^T^. The trout isolates exhibited the highest 16 S rRNA sequences similarities with *C. shigense* GUM-Kaji^T^ (99.2-99.8 %), percentages higher than the 99 % sequence similarity used as the criterion for species identification [19]. In addition, most of the phenotypic characteristics of the *C. shigense* trout isolates were consistent with the current description of this species based solely in the strain DSM 17126^T ^[20], which support the identification based on 16 S rRNA gene sequencing. Despite the overall phenotypic similitude, some differences were found between the *C. shigense* trout isolates and the type strain *C. shigense* CCUG 61059^T^. Thus, trout isolates reduced nitrate, assimilated citrate but not mannitol and produce the enzyme valine arylamidase but not the enzymes ester lipase C8 and β-glucosidase, while the type strain of *C. shigense* CCUG 61059^T^ gave opposite reactions for these traits.

Widely spread microorganisms are usually genetically heterogeneous [21]. Members of the genus *Chryseobacterium* are isolated from a wide range of environments [1] and therefore, it could be expected to be genetically diverse. However, trout isolates exhibited undistinguishable RAPD fingerprints indicating that they represent a single strain. This fact, together with their isolation in pure culture from internal organs might suggest a clinical significance; however the role of *C. shigense* as the causative agent of the disease episodes in trout cannot be undoubtedly established in absence of experimental infections trials.

Some members of the family *Flavobacteriaceae,* as *Chryseobacterium,* usually are opportunistic pathogens [4], because they require the existence of different predisposing factors such as coinfections with other bacteria or virus [7] or other environmental or husbandry sources of stress. No infestations or viral infections were detected previously to the disease episodes, but these usually occurred after transportation of rainbow trout fry from the hatchery to fish farm, or in tanks with elevated stock densities. These circumstances represent stressful conditions for fish [22] and might have increased the susceptibility of fry trout to infection.

Several species of *Chryseobacterium,* such as *C. piscicola *[8], *C. chaponense *[11], and *C. shigense* in this study, have been isolated from diseased fish in which *F. psychrophilum* infections were initially suspected. Although there are no clear evidences for considering these species as consistent pathogens for fish, they should be considered for a differential diagnosis in those cases with a suspicious of *F. psychrophilum* infection. Table 1 shows some phenotypic characteristics that can be useful for their differentiation.

**Table 1:** **Phenotypic characteristics**^**a**^**that can be useful to differentiate the species*C. shigense*,*C. piscicola,*C.*chaponense*and*F. psychrophilum***

**Characteristic**	***C. shigense***	***C. piscicola***	**C.*chaponense***	***F. psychrophilum***^***b***^
Growth at/with:				
37 C	-	-	+	-
3.0 % NaCl	w	+	-	-
Flexirubin-type pigment	+	+	-	+
Hydrolysis of:				
Casein	+	-	-	+
L-tyrosine	+	-	-	V
Gelatin	+	+	-	+
Starch	+	+	-	-
DNA	-	w	-	-
Enzyme activity:				
α-glucosidase	-	+	+	V
β -glucosidase	-	+	+	V
β-galactosidase	-	-	-	V

^a^ Data for *C. shigense* are taken from this study, for *C. piscicola* from reference Ilardi et al. [8], for *C. chaponense* from reference Kämpfer et al. [11] and for *F. psychrophilum* from references Bernardet et al. [23], Cipriano and Holt [18] and Hesami et al. [24]. ^b^*F. psychrophilum* differs also from *C. shigense* by its inability to utilize glucose as sole carbon source, to hydrolyze aesculin and to produce a brown diffusible pigment on tyrosine agar. +, Positive reaction; -, negative reaction; V, variable reaction; w, weak reaction.

Since the initial description of *C. shigense* from a fresh lactic acid beverage in Japan [20], no further isolations of this species have been reported. Consequently, the isolation of *C. shigense* from trout shows that it can also occur in a very different habitat. To our knowledge, this is the first description of the isolation of *C. shigense* from clinical specimens.

## Conclusions

In this work we describe by first time the recovery of *C. shigense* from clinical specimens in trout, showing that it can also occur in a very different habitat to fresh lactic acid beverage where it was initially isolated.

## Authors’ contributions

LZ carried out the phenotypic and genetic analyses and participated in the analysis of the data and drafting of the manuscript. AIV participated in the design of the study, in phylogenetic analysis and drafting of the manuscript. MAP participated in the recovery of clinical specimens. LD participated in the design of the study. JFJG conceived the study and participated in its design, drafting and coordination. All the authors read and approved the final manuscript.
